# Mr. Bernard Hollander

**Published:** 1907-10-19

**Authors:** Bernard Hollander

**Affiliations:** 35a, Welbeck Street, Cavendish Square, W.


### MR. BERNARD HOLLANDER.

Sir,—I wrote to you yesterday that through no fault of mine a sensational account of a lecture by me has appeared in the general Press. I see to-day extracts from it put together as if there had been an interview.

I cannot describe how profoundly sorry I am that this discreditable sensation has occurred, and wish to tender my humblest apologies, through your medium, to all members of my profession. I may hold singular individual views, but I have no desire to ventilate medical matters through the lay Press. Yours truly,

Bernard Hollander.

35a, Welbeck Street, Cavendish Square, W.
October 10, 1907.

